# Can an amino acid mixture alleviate gastrointestinal symptoms in neuroendocrine tumor patients?

**DOI:** 10.1186/s12885-021-08315-4

**Published:** 2021-05-20

**Authors:** Aman Chauhan, Satya Das, Rachel Miller, Laura Luque, Samuel N. Cheuvront, James Cloud, Zach Tarter, Fariha Siddiqui, Robert A. Ramirez, Lowell Anthony

**Affiliations:** 1 Division of Medical Oncology, University of Kentucky, Lexington, KY USA; 2 Markey Cancer Center, University of Kentucky, 800 Rose Street CC402, Lexington, KY 40536 USA; 3 Division of Medical Oncology, Vanderbilt University Medical Center, Nashville, TN USA; 4 Science & Technology, Entrinsic Bioscience Inc., Boston, MA USA; 5 School of Medicine, University of Kentucky, Lexington, KY USA; 6 Division of Oncology Ochsner Health System, New Orleans, LA USA

**Keywords:** Neuroendocrine tumors, Amino acid oral rehydration solution, Diarrhea, Excess bowel movements

## Abstract

**Background:**

Neuroendocrine tumors, although relatively rare in incidence, are now the second most prevalent gastrointestinal neoplasm owing to indolent disease biology. A small but significant sub-group of neuroendocrine tumor patients suffer from diarrhea. This is usually secondary to carcinoid syndrome but can also be a result of short gut syndrome, bile acid excess or iatrogenic etiologies. Recently, an amino acid based oral rehydration solution (enterade Advanced Oncology Formula) was found to have anti-diarrheal properties in preclinical models.

**Methods:**

A retrospective chart review of all NET patients treated with enterade AO was performed after IRB approval.

**Results:**

Ninety-eight NET patients who had received enterade AO at our clinic from May 2017 through June 2019 were included. Patients (*N*=49 of 98) with follow up data on bowel movements (BMs) were included for final analysis. Eighty-four percent of patients (41/49) had fewer BMs after taking enterade AO and 66% (27/41) reported more than 50% reduction in BM frequency. The mean number of daily BMs was 6.6 (range, 320) at baseline before initiation of therapy, while the mean number of BMs at 1 week time point post enterade AO was 2.9 (range, 011).

**Conclusions:**

Our retrospective observations are encouraging and support prospective validation with appropriate controls in NET patients. This is first published report of the potential anti-diarrheal activity of enterade AO in NET patients.

## Background

Neuroendocrine tumors (NETs) are rare and unique slow growing tumors that can originate from varied organ sites [1]. The prevalence of NETs has increased in the United States over 6-fold from 1973 to 2012, primarily due to improved diagnostics for early-stage disease and potency of systemic treatments leading to improved survival in the metastatic setting [2, 3]. NETs are classified as non-functional or functional tumors; non-functional tumors do not secrete hormones, while functional, non-pancreatic, NETs primarily secrete serotonin, resulting in carcinoid syndrome [1]. Approximately 1050% of patients with NETs will develop carcinoid syndrome [4,7] and its associated symptoms of flushing and frequent, often explosive, watery diarrhea (6278%) [4, 5, 8]. Carcinoid syndrome diarrhea can be distressing with many patients reporting bowel movements (BM) ranging from two to 30 or more per day [9]. Patients with NETs may also have moderate to severe diarrhea due to other etiologies including toxicity from chemotherapy, radiation and sequelae of gastrointestinal surgery [10,17].

Diarrhea and BM frequency in functional NET patients is typically managed with somatostatin analogs and telotristat ethyl (tryptophan hydroxylase inhibitor). Over-the-counter anti-diarrheal medications are also often utilized for breakthrough diarrhea. However, it is not uncommon for NET patients to continue to have persistent debilitating diarrhea despite treatment with multimodal agents.

We examined the potential for enterade Advanced Oncology (AO) Formula to reduce BM frequency in NET patients. Enterade AO consists of a unique blend of five amino acids (Valine, Aspartic Acid, Serine, Threonine, Tyrosine) selected for their ability to restore bowel absorption and integrity [18]. Enterade AO additionally contains electrolytes and flavors. Preclinical data suggest that enterade AO can restore enteral integrity following radiation-induced gut damage in mice [15, 17, 19]. We previously reported our anecdotal experience with enterade AO in a cancer patient who noted significant clinical improvement in gastrointestinal symptoms [20]. Non-toxic and inexpensive enteral nutritional therapies for decreasing diarrhea and BM frequency in NET patients, regardless of cause, can significantly improve patient quality of life and reduce patient and hospital costs [20]. Because of these observations, we elected to review our experience with enterade AO in reducing BM frequency in NET patients undergoing cancer treatments.

## Methods

### Ethics statement

Retrospective chart review was conducted after appropriate University of Kentucky Institutional Review Board (IRB) approval.

### Objective and hypothesis

A retrospective chart review of all NET patients treated with enterade AO under supervision of a registered dietitian in an oncology clinic setting was performed. All NET patients managed at Markey Cancer Center are screened for chronic diarrhea that is co-managed by medical oncology and registered dietitians. Interventions include pharmacologic and counseling measures focused on diet and nutrition. Any patients experiencing 4 or more stools were instructed to consume one 8oz. bottle of enterade AO twice daily, 30min before meals or 1h after meals for at least 1week. Patients were provided a one-week supply of samples (supported by Lockey Foundation Philanthropic Grant to Markey NET Clinic). If patients noted improvement in diarrhea, they were able to continue enterade AO by buying the product over the counter. Most patients were followed up at 1week over the phone to determine the number of BMs per day, and if the patient had any adverse side-effects from enterade AO. Those who had no follow up at 1 week or were lost to follow up were not included in this analysis.

The clinical data were retrospectively reviewed with a primary objective of evaluating change in BM frequency from before the trial of enterade AO suppression compared to after. The hypothesis was that enterade AO, when combined with standard supportive care, would improve small bowel absorption, leading to a reduction in BM frequency.

Medical records were also reviewed to obtain demographic data, information on the tumor type and location, histopathology, frequency of diarrhea, and use of somatostatin analogs.

### Statistical analysis

Data were analyzed using descriptive statistics to generate means and ranges. Robust linear regression was used to estimate the reduction in BM frequency afforded by use of enterade AO as a function of initial severity of diarrhea. All statistical analyses were performed using Prism GraphPad, version 8.0.

## Results

### Patient characteristics

We identified 98 NET patients who had received enterade AO at our clinic from May 2017 through June 2019. Patients (*N*=49 of 98) with follow up data on BMs were included for final analysis. The average age was 61years with a range of 33 to 84years. Thirty-seven (75%) patients possessed gastroenteropancreatic NETs, 8 possessed bronchial NETs, 1 possessed a gynecological NET, and 3 possessed NETs of unknown primary origin. Twenty-eight patients (57%) had a history of prior bowel resection either for primary NET resection or debulking. Twenty-eight patients (57%) were on somatostatin analogs at the time of initiation of enterade AO.

### Antidiarrheal efficacy of enterade AO

Eighty-four percent of patients (41/49) had fewer BMs after taking enterade AO and 66% (27/41) reported more than 50% reduction in BM frequency. The mean number of daily BMs was 6.6 (range, 320) at baseline before initiation of therapy, while the mean number of BMs at day 7 after starting enterade AO was 2.9 (range, 011).

**Fig. 1:**
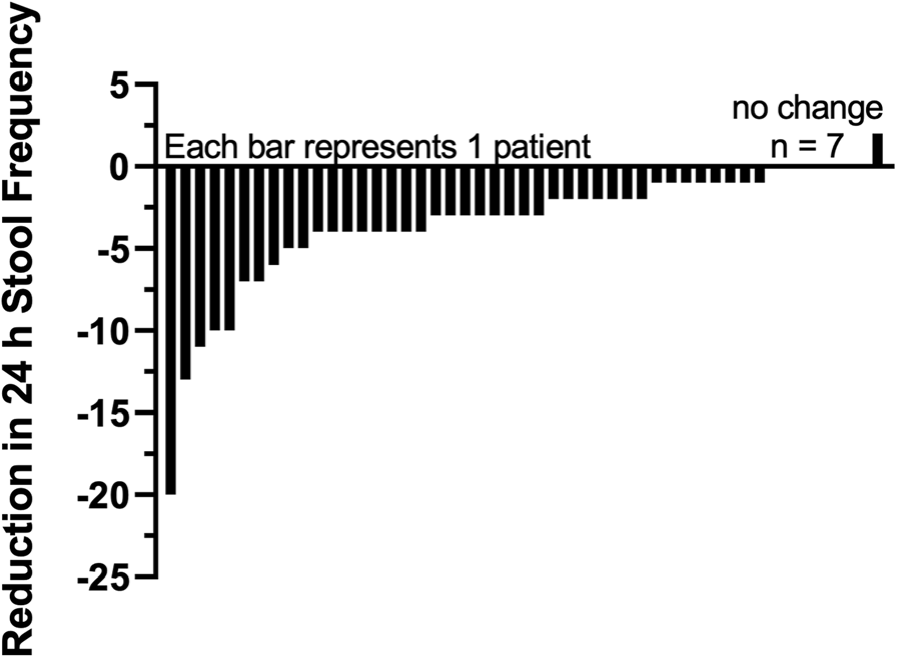
Plot of reduction in daily bowel movement frequency after use of enterade for 1-week for *n*=49 patients. Data are sorted and ordered from largest to smallest benefits to illustrate the effect magnitude and individual variability. Source: Author

The average time to improvement was 4.3days. Five patients had a marked decrease in BMs during the study and their diarrhea completely resolved (zero diarrheal BMs) by the end of the study. One patient reported the side effect of constipation. There were no other adverse reactions reported. Figure1 Illustrates the reduction in daily BM frequency for individual NET patients after using enterade for at least 1-week. Data are sorted and ordered from largest to smallest benefits to illustrate the effect magnitude and individual variability for *n*=49 patients.

## Discussion

The principal finding from this retrospective chart review of NET patients experiencing life-limiting BM frequency was that consumption of enterade AO resulted in a reduction in diarrhea, and that patients experiencing the most severe number of BMs appeared to derive the most benefit.

Traditionally, diarrhea in NET patients is managed with somatostatin analogs, anti-motility agents, and opioids. Nevertheless, some patients can continue to have persistent debilitating diarrhea despite the utilization of multi-modal agents. There are limited treatment options for controlling diarrhea in this population, especially for patients with severe, uncontrollable diarrhea. Somatostatin analogs are reported to be 6584% effective in decreasing the frequency of BMs [21]. However, it is common for patients to become refractory to somatostatin analogs. Telotristat ethyl, the first and only drug approved by the United States Food and Drug Administration for carcinoid syndrome diarrhea refractory to somatostatin analogs, reduced diarrhea in 44% of NET patients [22]. While telotristat is a significant advance for carcinoid NET patients with diarrhea, there are still NET patients with both functional syndrome as well as non-syndromic diarrhea which can further benefit from optimization of anti-diarrheal strategies.

**Fig. 2:**
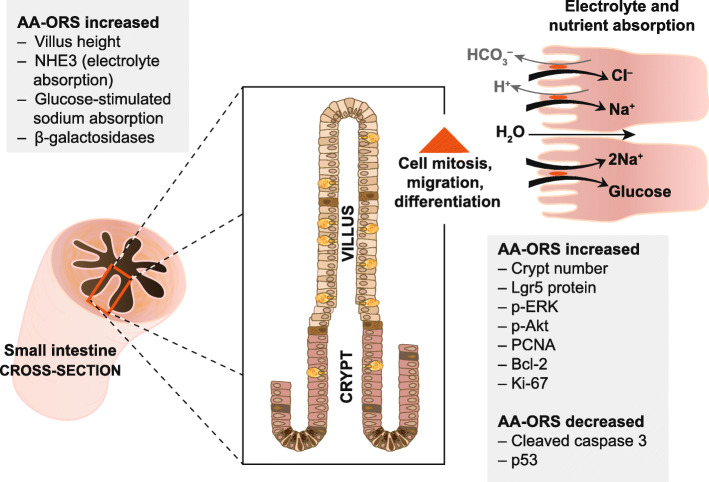
Illustration highlighting mechanisms of action of enterade Advanced Oncology Formula. Enterade modulates intestinal transmembrane proteins to promote intestinotrophic villus regrowth, increase sodium and water absorption, decrease chloride and bicarbonate secretion, and reduce intestinal paracellular permeability. See Yin et al., 2016 for further details. Source: Author

Enterade is a proprietary blend of five amino acids (threonine, valine, serine, tyrosine, tryptophan) that acutely restores water and electrolyte losses by facilitating intestinal sodium and water transport with similar stoichiometry to glucose [23], but without stimulating chloride secretion like glucose [24] Used prophylactically and chronically as a treatment, enterade modulates intestinal transmembrane proteins to promote intestinotrophic villus regrowth, increase sodium and water absorption, decrease chloride and bicarbonate secretion, and reduce intestinal paracellular permeability [24] (Fig.2). The benefits of enterade have been demonstrated by improvements in body weight maintenance and survival in mice with radiation enteritis and improved diarrhea outcomes in oncology patients suffering from toxic gut syndrome [24]. The potential benefits of a non-toxic and inexpensive enteral nutrition therapy like enterade AO for effectively treating diarrhea in NET patients looks promising and should be explored further in a prospective study. Limitations of our current study include lack of control group and a heterogenous patient population, lack of designated follow up and lack of a uniform outcome measurement. As mentioned previously, diarrhea in NET patients can be due to carcinoid syndrome, short gut syndrome, bacterial overgrowth, bile acid colitis, steatorrhea etc. Our study was not controlled for potential confounders and did not consider the effect of supportive care medications in addition to enterade AO. Furthermore, 50% of patients in the initial cohort did not have adequate follow up. It is possible that these patients derived less benefit from enterade AO, and this loss to follow up introduced selection bias. Also, as the post-treatment BM frequency assessment was carried out approximately at 1week, some potential heterogeneity could have been observed around time of outcome assessment. Despite these limitations, our early clinical observation suggests potential anti-diarrheal activity of enterade AO and warrants validation of the product in a well-controlled prospective clinical trial.

Our findings support further investigations of enterade AO for diarrhea mitigation in NET patients. This is not entirely surprising as the enterade AO mechanisms of action include hyper-absorptive, anti-secretory, barrier tightening, and villi proliferation effects within intestinal epithelia [15, 17, 19] which directly combat many of the pathophysiological effects of disease and treatment (radiation and chemotherapy) [25, 26] (Fig. 2). Although the placebo effect and biased reporting of favorable outcomes cannot be ruled out as factors which influenced outcomes in our analysis, the clinical outcomes observed are promising and consistent with rigorous pre-clinical anti-diarrheal data; these findings warrant further affirming prospective research.

## Conclusion

Eighty-four percent (41/49) of NET patients reported BM reduction with enterade AO while 66% (27/41) reported more than 50% reduction in BM frequency. In conclusion, our retrospective observations are encouraging and support prospective validation in NET patients. This is first published report of potential anti-diarrheal activity of enterade AO in NET patients. There are two ongoing prospective phase 2 studies (NCT02919670, NCT03722511) which are currently evaluating the ability of enterade AO to reduce BM frequency and relieve gastrointestinal symptoms in this population.

## Data Availability

The datasets used and/or analyzed during the current study are available from the corresponding author on reasonable request.

## Abbreviations

AO: Advanced Oncology
BM: Bowel movement
NET: Neuroendocrine tumor
